# Hemophagocytic lymphohistiocytosis with immunotherapy: brief review and case report

**DOI:** 10.1186/s40425-018-0365-3

**Published:** 2018-06-05

**Authors:** Masood Sadaat, Sekwon Jang

**Affiliations:** 1 Inova Center for Personalized Health, Inova Schar Cancer Institute, 3225 Gallows Rd, 7th Floor, Tower D, Fairfax, VA 22031 USA; 2 Inova Center for Personalized Health, Inova Schar Cancer Institute, 8505 Arlington Blvd Suite 140, Fairfax, VA 22031 USA

**Keywords:** Hemophagocytic lymphohistiocytosis, HLH, Immune checkpoint inhibitors, Checkpoint blockade, Pembrolizumab, Ipilimumab, Nivolumab, iRAE, Natural killer cells, sCD163, PD-1, PDL-1, Macrophage activation syndrome, Cytokine release syndrome, CAR T, BiTe

## Abstract

**Background:**

Hemophagocytic Lymphohistiocytosis (HLH), a rare but potentially fatal syndrome of immune hyperactivation, may be an under-recognized immune-related adverse event (irAE). Unlike other irAEs, HLH triggered by immune checkpoint blockade is not well described; no particular diagnostic guidelines and treatment regimens exist. The HLH-2004 criteria remain as the common diagnostic guide. For the treatment of HLH, various combinations of chemotherapeutic, immunosuppressive and glucocorticoid agents are used.

**Case presentation:**

We report a case of HLH in a 58-year-old metastatic melanoma patient who was undergoing immune checkpoint blockade with pembrolizumab, a programmed cell death-1 (PD-1) receptor inhibitor. The patient presented with fever, upper normal sized spleen, anemia, thrombocytopenia, hypertriglyceridemia, hyperferritinemia, reduced NK cell activity and elevated sCD163 levels, fulfilling the Histiocyte Society HLH-2004 diagnostic criteria. Our patient was successfully treated with oral prednisone (1 mg/kilogram/day), suggesting that HLH from immune checkpoint inhibitors may respond to steroids alone.

**Conclusion:**

Early diagnosis and treatment of HLH are critical to avoid progressive tissue damage, organ failure and possibly death. HLH should be suspected in clinical presentations with fever, cytopenias and hyperinflammatory markers. HLH in the setting of immune checkpoint blockade may be treated with steroids only but further evidence is required.

## Background

As immunotherapy continues to evolve and show promise in the treatment of various cancers, timely diagnosis and effective management of immune-related adverse events (irAEs) become more important. Some irAEs such as hemophagocytic lymphohistiocytosis (HLH) can be systemic and deadly.

Hemophagocytic Lymphohistiocytosis refers to a potentially fatal clinical syndrome of hyperinflammation and progressive immune-mediated organ damage due to over-stimulated but ineffective immune response [1]. Clinical features usually include those listed in HLH-2004 criteria (Table 1) [2, 3].

**Table 1:** Histiocyte Society HLH-2004 diagnostic criteria [2, 3]

The diagnosis HLH requires that either 1 or 2 below are fulfilled:	
(1) A molecular diagnosis consistent with HLH: Pathological mutations of *PRF1, UNC13D, STXBP1, RAB27A, STX11, SH2D1A, or XIAP*	
OR	
(2) Diagnostic criteria for HLH fulfilled (5 out of the 8 criteria below):^a^	
(A) Initial diagnostic criteria	
• Fever 38·5 °C or more	
• Splenomegaly	
• Cytopenias (affecting at least 2 of 3 cell lineages in the peripheral blood):	
○ Hemoglobin < 90 g/L (in infants < 4 weeks: hemoglobin < 100 g/L)	
○ Platelets < 100 × 10^9^/L	
○ Neutrophils < 1.0 × 10^9^/L	
• Hypertriglyceridemia and/or hypofibrinogenemia:	
○ Fasting triglycerides ≥3.0 mmol/L (i.e., ≥ 265 mg/dL)	
○ Fibrinogen ≤1.5 g/L	
• Hemophagocytosis in bone marrow or spleen or lymph nodes or liver	
(B) New diagnostic criteria	
• Low or absent NK-cell activity	
• Ferritin ≥500 mg/L	
• Soluble CD25 (i.e., soluble IL-2 receptor) ≥ 2400 U/mL^b^	

^a^Supportive criteria include neurologic symptoms, cerebrospinal pleocytosis, conjugated hyperbilirubinemia and transaminitis, hypoalbuminemia, hyponatremia, elevated D-dimers, and lactate dehydrogenase. The absence of hemophagocytosis (in the bone marrow) does not exclude a diagnosis of HLH

^b^New data show normal variation by age. Level should be compared with age-related norms

In primary HLH, which manifests mainly in childhood, mutations occur in genes that encode essential protein components of the cytotoxic machinery of T lymphocytes and natural killer (NK) cells. Altered genes involved in immunodeficiency syndromes also constitute the causes of primary HLH. Acquired HLH, with or without genetic disorders, may be due to infectious (bacterial, fungal, parasitic and viral) or non-infectious etiologies and triggers (malignancies, autoimmune disorders, and drugs) [3].

The exact pathophysiology of HLH varies depending on the cause and trigger [4]. Based mainly on the pathophysiology of primary HLH, defective granule-mediated cytotoxicity of cytotoxic T lymphocytes (CTLs) and natural killer (NK) cells is considered the main abnormality that causes HLH. Since CTLs and NK cells cannot insert perforin channels into the membranes of antigen presenting cells (eg, macrophages and histiocytes) and deliver granzymes, osmolysis and apoptosis of the antigen presenting cells do not occur. With persistent antigenic stimulation of CTLs and NK cells by the antigen presenting cells, an abundant release of cytokines ensues. The cytokine storm creates a systemic inflammation that can cause tissue destruction, progressive organ failure and death. Activated macrophages may engulf blood cells and create hemophagocytosis [5], the pathologic feature of HLH.

Malignancy-associated HLH (M-HLH) refers to HLH that occurs due to malignancy or happens during cancer treatment. The incidence of M-HLH is cited as 1% with a median survival of 1.5–2.5 months [6]. The relatively recent use of immunotherapies (eg, immune checkpoint inhibitors, bispecific mono-clonal antibody and bispecific T-cell engagers [BiTe]; chimeric antigen receptor T-cell therapies [CAR T], dendritic vaccines, and immunomodulatory drugs) in the treatment of various cancers may add to the effects of malignancy on immune homeostasis [6, 7].

Removal of normal control on important immune system pathways such as PD-1, PDL-1 and CTLA-4, may result in unique irAEs (eg, dermatitis, ophthalmological disorders, endocrinopathies, myocarditis, pericarditis, vasculitis, colitis, hepatitis, nephritis and pneumonitis) [8]. These irAEs are fairly well described and usually, manageable with the administration of high-dose glucocorticoids [9]. The incidence of HLH due to immunotherapy, however, has been rarely reported. The few reported cases had different diagnostic and therapeutic approaches with variable outcomes. Here we report a case of HLH triggered by pembrolizumab that was treated successfully with high-dose glucocorticoids. We also present a brief review of literature regarding the HLH cases due to immunotherapy, the dilemmas in diagnosing HLH, and the therapeutic hurdles in managing HLH.

## Case presentation

A 58-year-old man with metastatic melanoma was admitted to the hospital with 3-day history of high fever (up to 104.7 degrees Fahrenheit), nausea and arthralgias 31 days after receiving 6 doses of pembrolizumab (2 mg per kilogram of body weight). Initial workup revealed anemia (hemoglobin level, 9.9 g/dL [normal: 13 to 17 g/dL]) with low normal reticulocyte (0.6%, [normal: 0.5 to 2%]), thrombocytopenia (platelet level 101 × 103/microL [normal: 140-400 × 103/microL]), hypertriglyceridemia (triglycerides level, 309 mg/dL [normal: 0–149 mg/dL]), and marked elevation in ferritin (> 40,000.00 ng/mL [normal: 30–400 ng/mL]) and lactate dehydrogenase (2762 U/L [normal: 140–480 U/L]). Computed tomography depicted the size of spleen in upper normal (13 cm). Extensive lab and radiologic work up to identify any infectious agents was unremarkable. Study of natural killer (NK) cells showed decreased NK cell function. Soluble CD163 (sCD163) level was 6384 ng/mL (Reference range: 387–1785 ng/mL) (Table 2).

**Table 2:** Results of immunologic study^a^ of natural killer cells (NK cells) and sCD163 levels

A. Natural Killer (NK) Cell Function			
E:T Ratio	Result	Cytotoxicity	Reference Range
50:1	5%	Low	(> = 20)
25:1	2%	Low	(> = 10)
12:1	2%	Low	(> = 5)
6:1	1%		(> = 1)
NK Lytic Units	0.1	Low	(> = 2.6)
CD16/56% positive	4%	Low	(7–31)
Interpretation: Decreased NK cell function.			
B. sCD163 Level	6384 ng/mL		(387–1785 ng/mL)

^a^Immunologic study was conducted in Diagnostic Immunology Lab, Cincinnati Children’s Hospital

Patient was treated with high-dose glucocorticoids (oral prednisone administered at 1 mg per kilogram per day) within 24 h after admission. Rapid resolution of fever, nausea and gradual improvement in his arthralgia were noted. Patient’s anemia and thrombocytopenia improved after administration of glucocorticoids, therefore, we did not perform bone marrow biopsy. After 5 weeks of high-dose glucocorticoids, steroid dose was tapered over 7 weeks without recurring symptoms. Pembrolizumab was permanently discontinued. Patient achieved complete response in his metastatic melanoma for approximately 1 yr and then developed new metastases.

## Discussion

### A. Diagnostic dilemmas

Daver et al. state that less than 50% of adults with M-HLH received HLH-directed therapy because of lack of awareness and missed diagnosis of this condition in adult patients with malignancies in their center [1]. High morbidity and mortality due to HLH is partially attributed to delay in diagnosis. The rarity of the syndrome, non-specific and overlapping clinical picture with that of infection and sepsis, lack of validated criteria, and scarcity of diagnostic tools such as bone marrow biopsy, mutation testing and molecular assays are among the contributing factors. Online H-Score compiled by Fardet et al. [10] and a list of criteria developed by a panel of experts in the Delphi study [11] are examples of attempts to move towards precision and avoidance of delays in diagnosing HLH.

Our patient presented with fever, upper normal sized spleen, cytopenias affecting RBC and platelets, hypertriglyceridemia, high ferritin, low NK cell activity, thereby fulfilling the diagnostic criteria for HLH (Table 1). This patient was diagnosed with metastatic melanoma 2 years prior to receiving pembrolizumab, therefore it was unlikely that metastatic melanoma itself triggered HLH. In the absence of an infectious cause, we concluded that HLH was related to pembrolizumab.

High fever and hyperferritinemia prompted us to consider HLH in our diagnostic workup. Hyperferritinemia is a non-specific marker indicating inflammatory, infectious, hepatocellular, renal, metabolic and many other processes. However, Carl et al. report that ferritinemia greater than 10,000 μg/L had a sensitivity of 90% and a specificity of 96% for HLH diagnosis [12]. Elevated ferritin levels are also implicated in prognosis. Grangé and colleagues cite that hyperferritinemia greater than 4780 μg/L predicted death with a positive predictive value of 93% but sensitivity of this finding was low (46%) [13].

Our diagnosis was confirmed by immunological studies showing decreased NK cell function and high sCD163 levels. As a hemoglobin-haptoglobin scavenger receptor, sCD163 is a lineage marker indicating macrophage expansion and hyperactivation [5], which may be a useful marker for diagnosis of HLH and related disorders [14]. Soluble IL-2 (also known as sCD25) is a helpful marker for diagnosis and disease severity but not widely available [15].

Bone marrow, lymph node, liver, spleen and even skin biopsies to detect hemophagocytosis and/or lymphocytosis can be used as supportive markers [16]. However, it is well known that the presence of hemophatocytosis or lymphocytosis is neither specific nor sensitive for HLH [1, 16]. Initial biopsy can be negative and repeat biopsies are required for follow up [17]. Daver and colleagues further argue that hemophagocytosis is not pathognomonic for HLH and may cause delayed or missed diagnosis [1]. With sufficient clinical and lab criteria, identifiable cause of HLH, and significant improvement in our patient’s condition, we forewent bone marrow biopsy [18]. However, our conservative approach may not be advisable for most patients unless expert panels identify criteria with defined diagnostic weight and value for M-HLH and other sub-types.

### B. Challenges in treatment

Specific HLH treatment guidelines based on randomized trials do not exist [3]. Management of initial phases of genetic and acquired HLH is similar and in addition to supportive therapy, specific treatment is aimed at control of cytotoxicity and immunomodulation. Based on HLH-94 and 2004 protocols, high-dose glucocorticoids, etoposide, methotrexate and cyclosporine are major components of the treatment regimen [19–21]. The efficacy and outcome of these protocols in adults have not been evaluated, although a global analysis of adult case reports indicates that etoposide-containing regimens improve survival in cancer and infection (71–75%) more than in autoimmune diseases (57%) [3]. Patients with genetic HLH may remain on maintenance therapy until allogeneic hematopoietic cell transplantation. In acquired HLH patients, the underlying cause needs to be treated [5]. Additionally, intravenous immunoglobulin therapy (estimated survival rate increase to 59–75%) and plasma exchange (survival of approximately 80%) have been tried. Biological treatments (eg, rituximab, infliximab and etanercept), anti-TNF drugs, anti-interleukin-1r (anakinra), anti-interleukin-6 (tocilizumab), and B-cell depleting drugs (rituximab, belimumab) have shown varying degrees of clinical efficacy in different HLH subtypes [1, 3]. Alemtuzumab, IFN-gamma inhibitor (NI-0501), and Janus kinase 1 (JAK1)/JAK2 inhibitor (ruxolitinib) are novel therapeutic agents either in trials or approved for HLH treatment [1].

The treatment of HLH requires careful analysis of underlying trigger, patient’s performance status, organ functions, and concomitant therapies. In case of M-HLH, the need for such analysis is even more crucial [1]. Treatment guidelines or trials are non-existent for management of M-HLH and HLH as an irAE. A prospective study of 63 patients in China used combination of chemotherapy with liposomal doxorubicin, etoposide and methylprednisolone as a salvage therapy for adult patients with refractory HLH. The regimen resulted in complete remissions in 27% and partial remissions in 49% of the patients [22].

### C. HLH due to immunotherapy

HLH has rarely been reported in patients receiving immune checkpoint inhibitors. Shah et al. reported a patient who developed HLH after 9 months of pembrolizumab treated with etoposide and dexamethasone [23]. In contrast, our case was successfully treated with glucocorticoids only suggesting that HLH from immune checkpoint inhibitors may respond to steroids alone.

Satzger et al. reported a 26-year-old female who developed HLH during treatment with nivolumab plus ipilimumab for metastatic melanoma. Their patient was treated with prednisone 2 mg/kg/day, which was tapered to 1 mg/kg/day after a week and mycophenolate mofetil was started 360 mg b.i.d with a subsequent increase to 720 mg b.i.d [24].

Malissen et al. reported 3 cases of “Macrophage Activation Syndrome” (a term traditionally reserved for HLH due to rheumatologic disorders, namely systemic juvenile idiopathic arthritis). The first patient, a 77-year-old male with metastatic melanoma who received Nivolumab therapy, died despite treatment with steroids (0.5 mg/kg). Second melanoma patient, 42-year-old male receiving ipilimumab after initial treatment with nivolumab, recovered with systemic corticotherapy and antibiotics. The third patient, 81-year-old male with Merkel Cell carcinoma developed MAS (HLH) shortly after the first dose of avelumab and died despite high-dose steroids [25].

Takeshita and colleagues reported hemophagocytic syndrome, interstitial pneumonia, and probable Stevens–Johnson syndrome in a 63-year-old woman with stage IV squamous non-small cell lung cancer with nivolumab. Their patient was treated with intravenous methylprednisolone [26], which is comparable to our patient.

T cell–engaging therapies such as the chimeric antigen receptor T (CAR T) cells and bispecific monoclonal antibodies / bi-specific T cell engagers (BiTEs) have been promising in the treatment of highly refractory B cell malignancies [27, 28]. T cell–engaging therapies harness the cell-mediated immune response to attack cancer cells without the involvement of the major histocompatibility complex. The main challenge of T cell-engaging therapies, however, has been toxicity. The most common toxicity is the cytokine release syndrome (CRS), a group of inflammatory symptoms due to cytokine elevations associated with T cell engagement and proliferation. CRS symptoms can range from mild and flulike to a severe inflammatory syndrome, including vascular leak, hypotension, pulmonary edema, and coagulopathy, which could lead to multi-organ failure, similar to HLH and MAS. Steroids and direct targeting of elevated cytokines have shown variable success in the treatment of CRS patients [28].

HLH has also been reported in 2 patients with multiple sclerosis after treatment with alemtuzumab [29], a humanized monoclonal antibody. The first patient, a mid-20s female, died despite treatment with IV corticosteroids and a molecular adsorbent recirculation system procedure. The second patient, a 28-year-old male, was treated with rituximab and corticosteroids.

## Conclusion

Our case and other reported cases indicate that HLH may occur due to immunotherapy. HLH, CRS and MAS have been reported with different immunotherapeutic agents. Significant morbidity and mortality due to these systemic inflammatory syndromes can impact the use and development of immunotherapeutic agents.

HLH can lead to progressive organ failure, hence early diagnosis and treatment are important. The case reports highlight the need for diagnostic and therapeutic guidelines as well as randomized trials to assess HLH management especially in adults with cancer. Optimal treatment for HLH likely caused by immune checkpoint inhibitors and other immunotherapeutic agents is not known. As suggested by our case and cases by other authors, early intervention with high dose steroids alone may be successful in the treatment of HLH from immune checkpoint blockade. Moreover, to avoid missed or delayed diagnosis, primary, emergency and critical care teams may benefit from enhanced knowledge regarding the diagnosis and treatment of irAEs, including HLH and CRS. HLH should be in the differential diagnosis of patients presenting with fever, cytopenias and any other signs or markers of a hyperinflammatory status.

## Abbreviations

BiTe: Bi-specific T cell engagers
CAR T: Chimeric antigen receptor T cells
CRS: Cytokine release syndrome
CTLA-4: Cytotoxic T lymphocyte-associated antigen-4
HLH: Hemophagocytic lymphohistiocytosis
irAE: Immune-related adverse event
MAS: Macrophage activation syndrome
PD-1: Programmed death-1
PDL-1: Programmed death ligand-1
